# Body-Machine Interfaces after Spinal Cord Injury: Rehabilitation and Brain Plasticity

**DOI:** 10.3390/brainsci6040061

**Published:** 2016-12-19

**Authors:** Ismael Seáñez-González, Camilla Pierella, Ali Farshchiansadegh, Elias B. Thorp, Xue Wang, Todd Parrish, Ferdinando A. Mussa-Ivaldi

**Affiliations:** 1 Department of Biomedical Engineering, Northwestern University, Evanston, IL 60208, USA; a-farshchiansadegh@northwestern.edu (A.F.); EliasThorp@u.northwestern.edu (E.B.T.); toddp@northwestern.edu (T.P.); sandro@northwestern.edu (F.A.M.-I.); 2 Sensory Motor Performance Program, Rehabilitation Institute of Chicago, Chicago, IL 60611, USA; camilla.pierella@northwestern.edu; 3 Department of Physiology, Physical Medicine and Rehabilitation, Northwestern University, Evanston, IL 60208, USA; 4 Department of Informatics, Bioengineering, Robotics, and Systems Engineering at the University of Genoa, 16145 Genoa, Italy; 5 Department of Radiology, Northwestern University, Evanston, IL 60208, USA; xue-wang@northwestern.edu

**Keywords:** body-machine interface, spinal cord injury, rehabilitation, white matter plasticity, diffusion tensor imaging, motor skill learning

## Abstract

The purpose of this study was to identify rehabilitative effects and changes in white matter microstructure in people with high-level spinal cord injury following bilateral upper-extremity motor skill training. Five subjects with high-level (C5–C6) spinal cord injury (SCI) performed five visuo-spatial motor training tasks over 12 sessions (2–3 sessions per week). Subjects controlled a two-dimensional cursor with bilateral simultaneous movements of the shoulders using a non-invasive inertial measurement unit-based body-machine interface. Subjects’ upper-body ability was evaluated before the start, in the middle and a day after the completion of training. MR imaging data were acquired before the start and within two days of the completion of training. Subjects learned to use upper-body movements that survived the injury to control the body-machine interface and improved their performance with practice. Motor training increased Manual Muscle Test scores and the isometric force of subjects’ shoulders and upper arms. Moreover, motor training increased fractional anisotropy (FA) values in the cingulum of the left hemisphere by 6.02% on average, indicating localized white matter microstructure changes induced by activity-dependent modulation of axon diameter, myelin thickness or axon number. This body-machine interface may serve as a platform to develop a new generation of assistive-rehabilitative devices that promote the use of, and that re-strengthen, the motor and sensory functions that survived the injury.

## 1. Introduction

Despite progress in the field of assistive technologies for people who suffered an injury to the spinal cord, most of the current devices to control computers and wheelchairs are set in place to require as little physical effort from the user as possible, and little attention has been paid to maintaining and strengthening the neural and muscular resources that survived the injury [1,2,3,4]. Spinal cord injury (SCI) leads to motor impairment, weakness, muscular and cortical atrophy and altered reflexes, and these have been shown to progress further with lack of exercise [5,6,7,8,9,10]. Even in individuals with injuries to the cervical spinal cord, some motor and sensory capacities may remain available in the upper body. Several studies have shown that using their remaining functions and keeping an active body is critical for people with SCI in order to avoid the collateral effects of paralysis and to potentially recover some of the lost mobility [5,6,7,11]. Therefore, it is crucial to develop the next generation of assistive-rehabilitative devices that promote learning through upper-body coordination.

Acquisition, retention and refinement of motor skills all rely on the capability of the nervous system to create new patterns of neural activation for accomplishing new tasks and for recovering lost motor functions [12]. Recent advances in neural imaging have allowed learning studies on juggling [13], balance [14] and body-machine interfaces (BMIs) by our group [15], to demonstrate motor skill learning-induced structural changes of cortical and subcortical areas in both gray matter and white matter by using diffusion tensor imaging (DTI). DTI non-invasively measures the direction and rate of water diffusion within tissue. White matter integrity is commonly measured by fractional anisotropy (FA), a normalized measure of the variance of the diffusion ellipsoid at each voxel [16]. FA values for white matter tissue have been shown to be affected by physiological parameters, such as axon diameter, axon number and myelin thickness [17].

Loss of somatosensory afference leads to functional cortical reorganization [18,19,20]. SCI has been shown to lead to spinal cord atrophy, cortical atrophy of primary and sensory cortex [8], descending motor tracts [9] and cortical reorganization of the sensorimotor system [8,10], and the degree of cortical reorganization is associated with the level of disability. Although the goal of most SCI treatments is to re-establish neural connections in order to restore motor function, it is unclear whether the anatomical and functional changes that follow injury can be reversed.

In this study, we investigated the rehabilitative effects and learning-induced changes in the brain white matter microstructure of people with high-level SCI after they practiced coordinated upper-body movements to control a computer cursor through a novel body-machine interface. Subjects learned to use the remaining ability of their shoulders and upper arms to perform movements that controlled a computer cursor to complete different related tasks. Complementary to [15], the purpose of this study was to identify changes in motor function and white matter by comparing clinical scores and FA values pre- and post-bilateral upper-body motor skill training in people with a high-level spinal cord injury. We started from the assumption that motor learning is likely to be associated with different brain reorganization in unimpaired subjects compared to subjects with tetraplegia, in consideration also of the greater need for the reorganization of motor functions in the latter group.

## 2. Methods

### 2.1. Experimental Setup

Five participants with tetraplegia (spinal cord injury at the C3–C6 level, complete American Spinal Injury Association (ASIA) Classification A, or incomplete ASIA B and C) and in the chronic stage (at least 6 months post-injury; characteristics in Table 1) gave their informed consent and were enrolled in this experiment, which was approved by Northwestern University’s Institutional Review Board (STU00104310). Participants performed visuo-spatial motor training tasks over twelve 1.5-h sessions: 2–3 sessions per week for a total of 4–6 weeks of training. An evaluation of the SCI participants’ upper-body ability was performed before, in the middle and after training with the BMI. An MRI brain scan was performed before and after training.

Magnetic resonance imaging (MRI) data for 14 control subjects had been previously collected in a different study approved by Northwestern University’s Institutional Review Board (STU00008159). Control subjects gave their consent for the data to be shared with our group.

### 2.2. Clinical Evaluation

An evaluation of the SCI participants’ upper-body ability was performed before, in the middle and a day after training with the BMI to test for changes on their physical state. We characterized their residual mobility to test if, from the clinical point of view, the training had a beneficial influence or, at least, no negative interference with the participants’ upper-body function. A psychological test (Beck’s Depression Inventory) and a measure of rehabilitation (Functional Independence Measure (FIM)) were performed during clinical evaluations to track the emotional state of participants.

Expert physical and occupational therapists from The Rehabilitation Institute of Chicago not involved in the study administered a Manual Muscle Test (MMT) following the procedure described in [21]. Each movement in the MMT is evaluated according to a 0–5 scale (0 = absent, 5 = normal). The movements evaluated for each shoulder were: scapular elevation, adduction and abduction, shoulder flexion, abduction, horizontal adduction and horizontal abduction and elbow flexion and extension.

A hand-held force sensor (Lafayette^®^ Manual Muscle Testing System, Lafayette Instrument Company, Lafayette, IN, USA) was used to measure the force exerted by each shoulder movement. Using an isometric force task, we asked participants to apply their maximum force against the sensor in different fixed positions two consecutive times. We recorded the larger force as their maximum force. The evaluated movements for each shoulder were: elevation, abduction, flexion, protraction, retraction, horizontal abduction, extension and horizontal adduction.

### 2.3. Magnetic Resonance Imaging

MRI data were collected for participants with tetraplegia within one week prior to the start of BMI training and within one week of the completion of training (one 4-min and one 8-min DTI session). MRI data were collected using a Prisma 3.0 Tesla (Siemens Medical Solutions, Erlangen, Germany) MR scanner with a 64-channel head/neck coil. Whole brain two shell diffusion tensor imaging data were collected in two separate acquisitions. The first had imaging parameters of 30 directions, TR/TE = 3500/65 ms, voxel resolution = 2 × 2 × 2 mm, 78 slices, multiband factor of 3, IPAT = 2, 6/8 partial Fourier, b value = 700 s/mm^2^, flip angle = 90°, and the second used identical imaging parameters, but with 64 directions and a b value of 2000 s/mm^2^. Each session collected a single image without any diffusion (b = 0 s/mm^2^).

### 2.4. Body-Machine Interface Calibration

**Figure 1:**
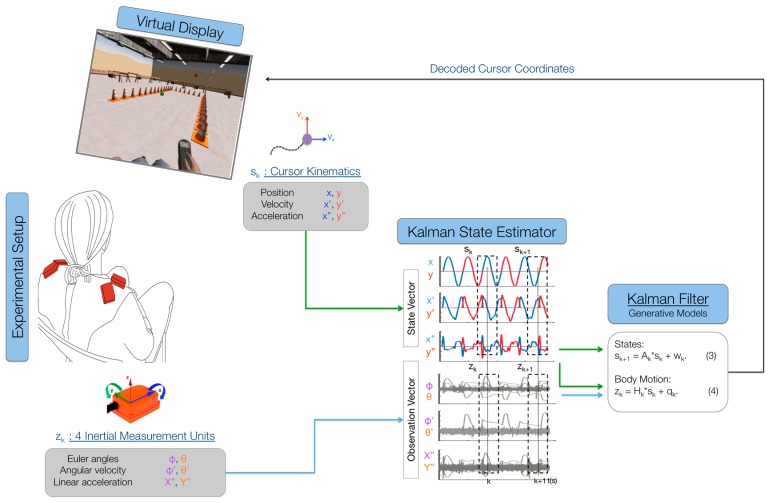
Kalman decoding of body motions into control signals for a virtual display. The participant sat in front of a computer monitor where the virtual cursor was displayed. Body motions were recorded using 3DOF inertial measurement units from Xsens and passed into the decoder as Euler angles, angular velocities and linear accelerations. Cursor position, velocity and acceleration were decoded from body motions using a Kalman filter. The body-machine interface then calculated the virtual display cursor’s position based on participant body movements and displayed it to the user via the computer monitor, thus closing the control loop for the user. The task of the participant was to use the virtual cursor to acquire a series of targets presented on the screen, type, play videogames and drive a virtual wheelchair.

The main purpose of the BMI was to map the high-dimensional body-signal vector of upper-body movements, measured by the IMUs, into a lower dimensional (2D) control vector. Participants were presented with a cursor on the computer monitor that moved with a pre-determined path (Figure 1). They were instructed to move their shoulders with the cursor as if they were already controlling it with their chosen movements. Participants were instructed to control horizontal cursor movements by moving their left shoulder up (elevation) and down (depression), respectively, and to control vertical cursor movements by moving their right shoulder up and down, respectively. The 2 cm-diameter cursor moved with a minimum-jerk velocity profile that resembles commonly-observed human point-to-point movement [22,23,24]. The cursor’s position history while moving from the center towards the right and back was governed by the function:

(1) $$x\left(t\right)=\;x_{0}+\left(x_{f}-x_{0}\right)\left(6\tau^{5}-15\tau^{4}+10\tau^{3}\right)$$

(2) $$y\left(t\right)=\;y_{0}+\left(y_{f}-y_{0}\right)\left(6\tau^{5}-15\tau^{4}+10\tau^{3}\right)$$

where τ = *t*/*t_t_*, $x_{0}$, $y_{0}$ are the initial cursor position coordinates at *t* = 0 and $x_{f}$ and $y_{f}$ are the final cursor position coordinates at *t* = *t_f_* [24]. The profile of cursor position, velocity and acceleration history during training is shown in Figure 1. The trajectory is a straight line between the center and final positions with a bell-shaped unimodal velocity profile. The total duration of each movement to the right and back lasted 4 s. A 6 × 6 cm box delimited the cursor’s movement range, so that participants knew where the cursor would stop moving and come back to the center and could plan to move their shoulders accordingly. The cursor moved to each of the four directions (up, down, right, left) six times for a total calibration time of 96 s.

While participants followed the cursor as if they were controlling it with their shoulders during the calibration phase, we logged body motion and cursor data at 50 Hz. The IMU’s Euler angles, angular velocities and linear accelerations were recorded at each time step *k* (every 20 ms) as the body observation in a 24-dimensional (2 angles, 2 velocities, 2 accelerations per each of 4 sensors) vector $z_{k}$. The position, velocity and acceleration of the cursor were recorded at each time step *k* as the cursor’s state $s_{k}$. Both data were fed into a Kalman state estimator to learn the matrices that relate body motions to cursor kinematics.

The purpose of the Kalman filter is to estimate the cursor’s state at every instant in time, based on the body-motion observations. The Kalman model assumes the state to be linearly related to the future state (next time step) by a stochastic linear function (Figure 1; Equation (3)). The model also assumes that the observation is linearly related to the state at each instant in time by another stochastic linear function (Figure 1; Equation (4)). Through these two assumptions, the calibration data from the cursor and the IMUs can be used to estimate the model’s matrices via least squares (for details, see [25,26,27]). After the model’s parameters have been estimated, the model encodes the body observation and cursor propagation, and participants can move their shoulders to control the 2D cursor on the screen. Participants were allowed to familiarize themselves with the control of the BMI before beginning the experiment (Figure 1). If participants had trouble controlling the map or if they did not feel comfortable with it, the calibration procedure was repeated.

### 2.5. Body-Machine Interface Training

Participants performed visuo-spatial motor training tasks over twelve 1–2 h sessions: 2–3 sessions per week for a total of 4–6 weeks of training. The first BMI training session consisted of calibrating the map several times for participants to understand how different movement combinations would affect the map. After participants chose one movement combination, they felt comfortable with, they performed one reaching test comprised of 24 center-out cursor movements to 8 peripheral targets. The second BMI training session consisted of calibrating the interface using the same movements they chose on the previous session and performing two reaching tests. The rest of the BMI training sessions consisted of a calibration phase followed by five tasks: (i) reaching; (ii) typing where participants controlled a cursor to type the pangram sentence “the quick brown fox jumps over the lazy dog” using a virtual keyboard; (iii) 3 blocks of a 2-min Pong-like game; (iv) 10 min of driving a wheelchair in a virtual environment; and (v) a repeat of the reaching in the first step. The games provided participants with an entertaining and motivating environment to practice and develop the upper-body dexterity that is required for successful navigation of the simulated wheelchair.

### 2.6. Data Analysis

#### Body-Machine Interface Performance Measures

Participant BMI performance was quantified by four performance measures. For each center-out trial, jerk, path length, error and movement time were computed. Jerk was computed as the time derivative of acceleration normalized by movement amplitude, duration and mean speed. This dimensionless measure of jerk has been shown to properly quantify common deviations from smooth movements [28], where a small jerk value would indicate a smooth, coordinated movement. Path length was computed as the sum of the Euclidean distance between consecutive cursor positions along each center-out reach trajectory, normalized by the straight-line distance between the starting and ending cursor positions. Path length quantifies movement “straightness” and “effectiveness”. A path length value of 1 would indicate the participant moved in a perfectly straight line from the center target to the peripheral target. Error was defined as the Euclidean distance between the cursor and target positions 1 s after movement initiation. Movement initiation was determined as the instant when the cursor’s velocity was above 10% of the velocity peak for that trial. Movement time was computed as the time between the target appearing on the screen, and 1 s before the participant completed the trial (target had to be held for one second). The movement time measure indicates the time that it took participants to complete the trial.

All performance measures were averaged over all trials to obtain one value per session or block for each participant. This resulted in a total of 12 values for SCI participants (12 sessions, averaged 1st and 2nd reaching blocks). Together, these performance measures allowed us to measure differences in cursor control proficiency among participants. Other performance measures were also computed (straight-line distance and aspect ratio), but they were highly correlated with the four listed above, so we used these four to characterize movement performance during the center-out reaching task.

Typing task performance was quantified by the total time it took to type the pangram. Pong performance was quantified by the number of times the participant was able to hit the ball with the paddle. Virtual wheelchair driving performance was quantified by two metrics. For each driving block, the number of checkpoints per minute and the number of collisions per minute were recorded.

In order to test for a learning effect, we performed a post-hoc paired *t*-test for the group of participants. We tested the null hypothesis that the mean difference between paired observations of the first vs. the last blocks was zero, with the alternative hypothesis that the mean was greater than zero. All statistical tests were repeated for each performance measure and allowed us to reject the null hypothesis at *p* < 0.05.

### 2.7. Diffusion Data Analysis

#### 2.7.1. FA, MD, RD and AD Estimation

Estimations of fractional anisotropy (FA), mean diffusivity (MD), radial diffusivity (RD) and axial diffusivity (AD), as well as all statistical and region of interest analyses were conducted with FSL Diffusion Toolbox (FDT, Version 5.0, FMRIB, Oxford, UK [29,30]). DTI data from both shells were concatenated and motion and eddy current corrected before the diffusion tensor at each voxel was calculated. Fractional anisotropy maps were derived based on the 3 eigenvalues of each tensor as described in [15].

#### 2.7.2. Tract-Based Spatial Statistics

Tract-based spatial statistics (TBSS) [31], a voxel-wise statistical analysis of FA data, was used to localize brain changes. All FA maps were aligned into a common standard space using the 2-mm isotropic FA standard-space template from 58 well-aligned good quality FA images (FMRIB58_FA) as a target and the nonlinear registration tool FNIRT [32]. A mean FA skeleton was created using the mean FA image across all participants thinned at a 0.2 threshold. This skeleton represents the centers of all tracts common to the group. Each participant’s aligned FA data were projected onto this skeleton, and the resulting data were fed into a voxel-wise cross-participant statistical analysis. In order to test whether SCI and control subjects had different FA values in regions commonly associated with motor control, fourteen regions of interest (ROIs) in white matter that are commonly involved in motor control—the anterior, superior, and posterior corona radiata, the cingulum and the anterior and posterior limbs of the internal capsule for each hemisphere, as well as the body and genu of the corpus callosum—were considered in this study. A mask containing the motor ROIs (Figure 5A) was created and applied to the FA skeleton using the Johns Hopkins University International Consortium of Brain Mapping ICBM-DTI-81 white-matter atlas in FSL [33]. A two-sample *t*-test with 5000 permutations was used to compare the mean FA, MD, RD and AD values between SCI and control participants. Permutation testing is non-parametric [34]; therefore, assumptions of normality and homoscedasticity were not necessary.

In order to test whether BMI training could induce changes in FA regions that showed degradation after the SCI, we created and applied a skeleton mask containing regions that showed significantly lower (*p* < 0.05, uncorrected) FA values in SCI subjects compared to controls. A paired *t*-test within these regions was used to compare mean values before and after training using the “randomize” command in FSL with 252 permutations and 5-mm FWHM Gaussian kernel smoothing. Cluster-like structures were enhanced using threshold-free cluster enhancement.

#### 2.7.3. Region of Interest Analysis

The fourteen motor ROIs were defined in the standard Montreal Neurological Institute (MNI) space, and all of the skeletonized maps of diffusion metrics were non-linearly transformed to the standard space for statistical analysis. Only data from the intersection of the MNI ROIs and the TBSS skeletons were analyzed. The means for non-zero FA, MD, RD and AD skeleton values within each ROI were calculated for each participant. The group difference between SCI and 14 non-impaired subjects, as well as the average change post-training relative to the baseline in the SCI group were calculated for each ROI. Each ROI was inverse transformed back to each participant’s native space for each scanning session in order to confirm that the voxels showing significant effects were indeed located within the white matter in each individual by visual inspection.

#### 2.7.4. Tractography Analysis

A tractography analysis was performed to better understand changes in the connectivity of the white matter fibers in regions that showed significant increases in FA values after BMI training in SCI participants. Tractography was performed by a deterministic fiber tracking algorithm [35] with a seeding region placed at the ROI of interest using DSI Studio software (7 March 2016 build, [36]). Following the TBSS results, we chose the left cingulum (TBSS result *p* < 0.05) ROI transformed to each subject’s native space as the seed region of interest. Constraints were placed on the deterministic tractography, such that the FA was >0.097, and the stepwise angular change was less than 60 degrees. Furthermore, tracks with a length less than 40 mm were discarded in order to limit local fiber influence. A total of 5000 seeds were placed. For each subject’s before and after image, the number, average length and volume were calculated for all tracts within the specified region of interest.

## 3. Results

### 3.1. Body-Machine Interface Performance

**Figure 2:**
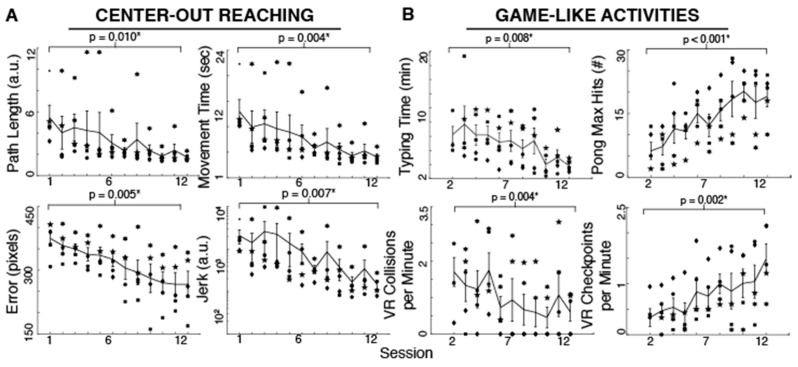
Spinal cord injured participant performance during 12 sessions of body-machine interface (BMI) computer tasks. (**A**) Individual subjects’ mean performance at each session is shown by a unique marker type. The lines represent the group averages (*n* = 5 participants, 48 trials per session) ±SEM (bars); (**B**) Participant’s performance during typing, Pong and virtual reality wheelchair driving. Horizontal lines on top of each graph represent the results for the one-sided paired *t*-test comparing the first to the last session. A star indicates a significant difference at *p* < 0.05.

Participants improved their reaching performance with training as evident in the different measures. Reaching movements became smoother (t_Jerk_ = 2.62, *p* = 0.030, 92.86% reduction), more accurate (t_Error_ = 4.60, *p* = 0.005, 28.77% reduction), straighter (t_PathLength_ = 3.76, *p* = 0.009, 68.30% reduction) and faster (t_MovementTime_ = 4.88, *p* = 0.004, 68.79% reduction) with 12 practice sessions (Figure 2). As shown in Figure 2, the typing time significantly decreased with training (t_TypingTime_ = 4.08, *p* = 0.007, 53.47% reduction). The maximum number of hits during Pong increased with practice (t_PongHits_ = 8.55, *p* < 0.001, 206.45% increase). Moreover, the effective VR driving speed as measured by checkpoints per minute increased and the number of collisions per minute decreased (t_CheckpointsPerMin_ = 6.33, *p* = 0.002, 341.33% increase, and t_CollisionsPerMin_ = 4.97, *p* = 0.004, 64.04% reduction) after 10–11 sessions (subjects started these tasks on their second session). Due to time limitations, two subjects began the typing, Pong and driving activities until their third session. We used their performance in that session as their first measure in the paired-sample *t*-test.

### 3.2. Upper-Body Ability

**Figure 3:**
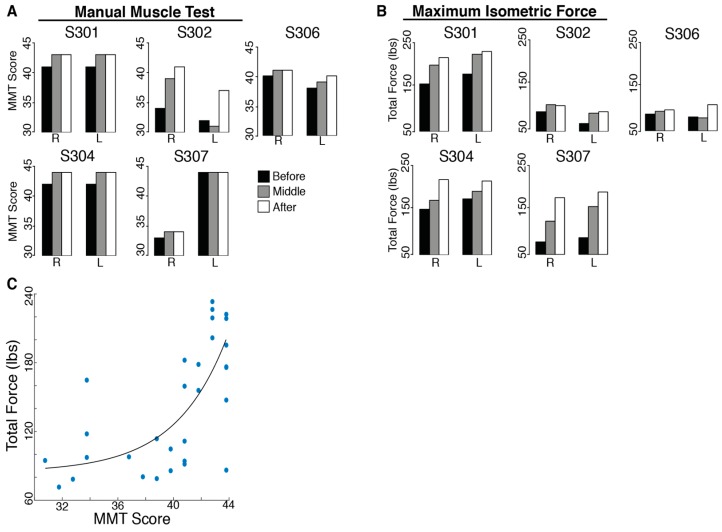
Upper-body ability of people with spinal cord injury performing 12 weeks of BMI training. (**A**) The total Manual Muscle Test (MMT) scores for the right (R) and left (L) shoulders before (black), in the middle (grey) and after (white) training; (**B**) The total maximum isometric force for the right and left shoulders and arms before, in the middle and after training; (**C**) Correlation between the MMT total score (*x* axis) and the total force score (*y* axis) measured with a hand-held force sensor (sum of the forces exerted by one shoulder and arm in the eight tested movements).

There was no evidence of harm being done by BMI practice. In fact, participants improved their upper-body ability with training. All SCI subjects had an initial MMT score far below the maximum possible score (45). The total MMT score improved significantly for all subjects after training (t_MMT_ = 2.40, *p* = 0.037; Figure 3). However, no individual evaluated muscles showed consistent increases for all subjects.

The total isometric force exerted by the subjects’ shoulders improved significantly for all subjects after 12 training sessions (t_ForceSensor_ = 3.56, *p* = 0.012). Individual maximum isometric force measurements that showed significant increases for all subjects included right shoulder flexion (t_RightFlexion_ = 4.50, *p* = 0.005, 22.94% increase), protraction (t_RightProtraction_ = 2.40, *p* = 0.037, 63.57% increase), retraction (t_RightRetraction_ = 2.68, *p* = 0.028, 118.41% increase), horizontal abduction (t_RightHzAbduction_ = 2.95, *p* = 0.021, 29.11% increase), extension (t_RightExtension_ = 2.73, *p* = 0.026, 87.00% increase) and horizontal adduction (t_RightHzAdduction_ = 2.27, *p* = 0.043, 47.87% increase), as well as left shoulder abduction (t_LeftAbduction_ = 2.61, *p* = 0.030, 40.48% increase), protraction (t_LeftProtraction_ = 4.07, *p* = 0.008, 62.65% increase), retraction (t_LeftRetraction_ = 5.14, *p* = 0.003, 113.51% increase), horizontal abduction (t_LeftHzAbduction_ = 3.78, *p* = 0.010, 62.68% increase) and extension (t_LeftExtension_ = 2.54, *p* = 0.032, 43.93% increase). The Manual Muscle Test is not a direct measure of strength; however, there was a moderate correlation (*r* = 0.685, *p* < 0.001) between the total MMT score for each subject’s shoulder and the total force measured with the force sensor (sum of the maximum forces for the sixteen tested movements).

**Figure 4:**
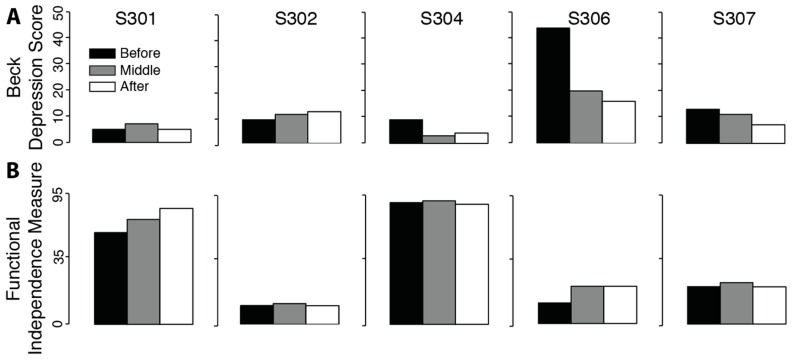
Psychological and functional evaluations for people with spinal cord injury performing 12 weeks of BMI training. (**A**) The total Beck Depression Inventory scores for each subject before (black), in the middle (grey) and after (white) training; (**B**) The total functional measure of independence for each subject before, in the middle and after training.

There was no significant change in measures of depression (Figure 4. t_Beck_ = 1.32, *p* = 0.129) or functional impairment (t_FIM_ = 1.47, *p* = 0.108). Positive effects for the Beck and FIM tests mean a reduction in measures of depression and impairment.

### 3.3. Upper-Body Ability Correlation with Behavior

Correlations between upper-body ability measures showing significant increases in maximum isometric force and the BMI measures are summarized in Table 2. Significant correlations between maximum isometric force and BMI performance were found between movement time and right shoulder protraction (*r* = 0.583, *p*_FDR_ = 0.038) and retraction (*r* = 0.651, *p*_FDR_ = 0.017) and left shoulder retraction (*r* = 0.779, *p*_FDR_ = 0.001); between Euclidean error and right shoulder protraction (*r* = 0.699, *p*_FDR_ = 0.015) and left shoulder retraction (*r* = 0.671, *p*_FDR_ = 0.006); between path length and right shoulder protraction (*r* = 0.565, *p*_FDR_ = 0.038) and retraction (*r* = 0.671, *p*_FDR_ = 0.017) and left shoulder retraction (*r* = 0.786, *p*_FDR_ = 0.001); and between jerk and right shoulder (*r* = 0.564, *p*_FDR_ = 0.038) and left shoulder (*r* = 0.682, *p*_FDR_ = 0.006) retraction.

### 3.4. Brain Differences in FA between SCI Subjects at Baseline and Controls (TBSS and ROI)

**Figure 5:**
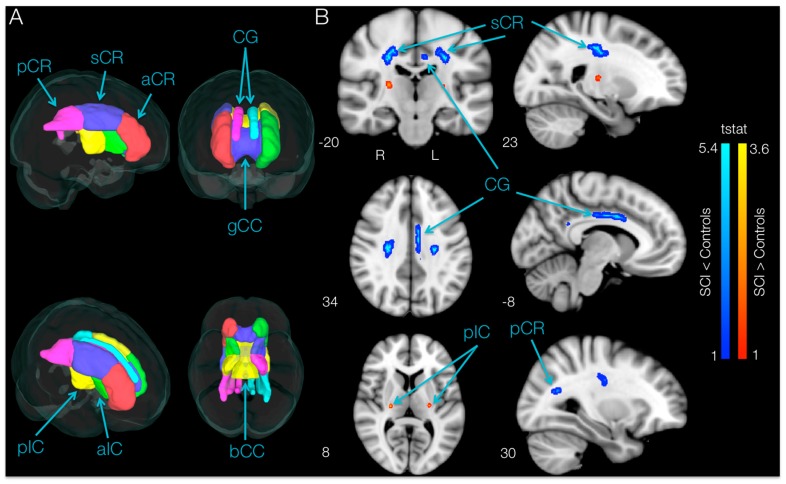
Regions showing lower fractional anisotropy (FA) in spinal cord injury (SCI) subjects compared to controls. (**A**) Brain regions associated with motor function used to perform tract-based spatial statistics (TBSS) and ROI analyses; (**B**) TBSS results. Regions showing significantly higher (red-yellow) and lower (blue-light blue) FA values in SCI versus control subjects overlaid over the standard Montreal Neurological Institute (MNI)152 *T*_1_-weighted anatomical scan (*p* < 0.05, uncorrected). The location of each slice in Montreal Neurological Institute space is shown at the lower left section. a-s-pCR, anterior, superior and posterior corona radiata; CG, cingulum; g-bCC, genu and body of corpus callosum; a-pIC, anterior and posterior limbs of internal capsule.

TBSS analysis of DTI indices within the motor areas (Figure 5A) in SCI participants compared to controls revealed areas with significantly lower (*p* < 0.05, uncorrected) FA values in SCI subjects bilaterally in the superior corona radiata, in the cingulum of the left hemisphere and in the posterior corona radiata of the right hemisphere. Moreover, TBSS analysis showed higher FA bilaterally in a small area of the posterior limb of the internal capsule in SCI subjects compared with controls.

#### ROI Results: SCI Subjects at Baseline vs. Controls

ROI analysis of white matter skeleton DTI indices within the motor areas of SCI participants revealed significantly lower (*p* < 0.05, uncorrected) FA values bilaterally in the anterior limb of the internal capsule (t_aIC_Left_ = 2.25, *p* = 0.019, 8.15% lower in SCI, and t_aIC_Right_ = 3.02, *p* = 0.004, 18.15% lower, respectively) when compared to controls. Lower FA values in SCI subjects on the left hemisphere occurred in the anterior corona radiata (t_aCR_ = 2.32, *p* = 0.016, 8.40% lower) and cingulum (t_CG_ = 2.22, *p* = 0.020, 16.04% lower; Figure 5B), whereas the only region with a lower FA value in SCI subjects in the right hemisphere was the superior corona radiata (t_sCR_ = 1.99, *p* = 0.032, 5.90% lower). The same regions had significantly higher MD, RD and AD. Moreover, the ROI analysis revealed no region had greater FA in SCI participants compared with controls.

### 3.5. Brain FA Changes after 12 Weeks of BMI Training in Subjects with SCI (TBSS and ROI)

**Figure 6:**
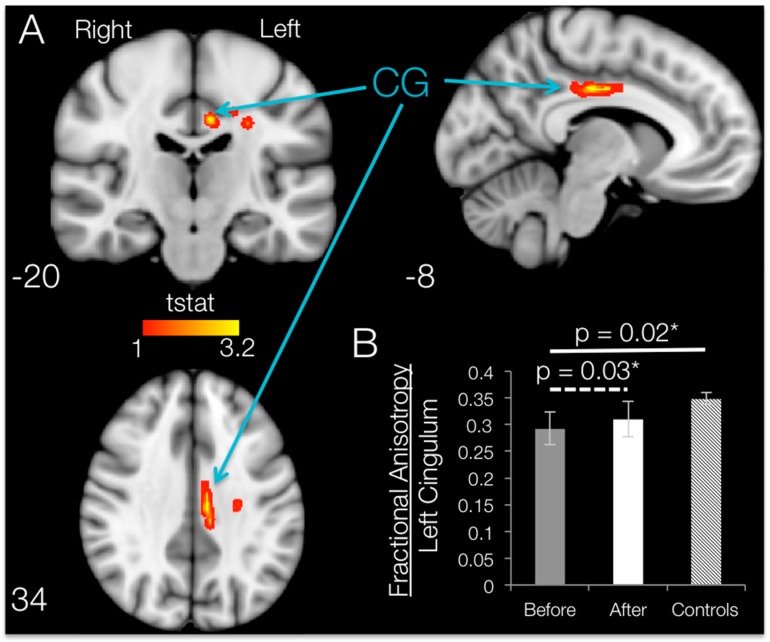
Regions showing increases in FA in SCI subjects after 12 weeks of BMI training. (**A**) Regions showing significantly higher FA (*p* < 0.05, uncorrected) in SCI subjects after BMI training overlaid over the standard MNI152 *T*_1_-weighted anatomical scan. The regions are thickened for visualization purposes only. The location of each slice in Montreal Neurological Institute space is shown at the lower left section. CG, cingulum; (**B**) FA of left cingulum before and after training for SCI subjects (dotted line, paired-sample *t*-test) and controls (solid line, two-sample *t*-test). Bars are the means ± SEM.

When comparing post- vs. pre-training mean FA maps after SCI within the regions that had shown degradation compared to controls, 12 weeks of BMI motor training significantly increased (*p* < 0.05, uncorrected) fractional anisotropy values localized to the white matter in the left hemisphere. Brain regions with significant increases in FA are shown in Figure 6. TBSS increases in fractional anisotropy were apparent for the left hemisphere cingulum, body of corpus callosum, superior corona radiata and/or posterior corona radiata.

#### ROI Results: SCI Subjects

ROI analysis on the damaged regions of interest (identified by using the Johns Hopkins University JHU white-matter atlas) revealed that the only ROI that showed a significant increase in FA following training was the cingulum, with an average increase of 6.02% (t_CG_ = 2.58, *p* = 0.031; Figure 6B). Although TBSS results show increases in FA in some of the other ROIs, whole-ROI increases for the left body of the corpus callosum (t_bCC_ = 1.46, *p* = 0.109, 0.77% increase), the superior corona radiata (t_sCR_ = 0.147, *p* = 0.445, 0.19% increase) and the posterior corona radiata (t_pCR_ = 0.836, *p* = 0.225, 1.14% increase) were not significant. No ROI had significant changes in MD, RD or AD values, and no brain area showed significant increases in FA from TBSS or ROI analyses.

### 3.6. Changes in FA Correlation with Upper-Body Ability and Behavior

We did not observe any significant correlations between the MRI measures that showed a significant increase after training, motor performance gain from the BMI and improvements in the clinical evaluation. The correlation coefficients and *p*-values for these results are shown in Table 3 and Table 4.

### 3.7. Tractography of Areas Showing Significant Increases in FA

**Figure 7:**
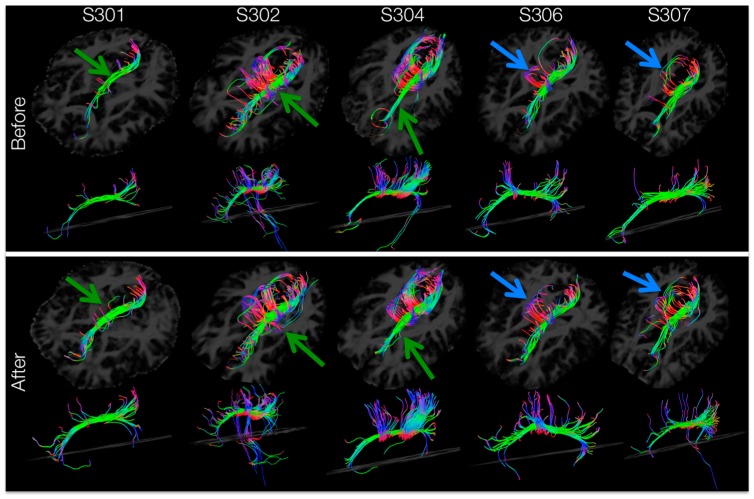
Tractography analysis of left cingulum for SCI subjects before and after BMI training. Tracts passing through the areas with significant (*p* < 0.05) increases in FA within the left-hemisphere cingulum are shown for each subject before (top), and after (bottom) training. Arrows highlight areas with an apparent increase in anterior-posterior (green) or inter-hemispheric (blue) tracts.

Tracts passing through the left-hemisphere cingulum ROI (masked for significant increases in FA) for each subject’s DTI image before and after training are shown in Figure 7. After training, tractography analysis resolved more tracts passing through the left cingulum connecting anterior to posterior areas of the brain (Figure 7, green arrows) and forming inter-hemispheric connections (Figure 7, blue arrows). Within the left cingulum, there were training-induced increases in resolved number of tracts (t_number_ = 3.063, *p* = 0.019), tract length mean (t_lengthMean_ = 4.420, *p* = 0.006) and tract volume (t_volume_ = 2.783, *p* = 0.025).

## 4. Discussion

This work demonstrates the feasibility of a body-machine interface that remaps the residual motor skills of people with high-level spinal cord injury into efficient patterns of control. This BMI can be adapted to fit each user’s residual motor ability. The focus of the study was to identify control signals for the operation of a two-dimensional device by unrestricted upper-body motions and to evaluate the rehabilitative effects of long-term practice using this BMI. Subjects with high-level spinal cord injury demonstrated their ability to operate the BMI from their first session and were able to improve their control with faster, smoother, straighter and more precise trajectories. By learning to use their body to guide the cursor, participants reached virtual targets on the screen, typed and played computer games. Moreover, participants were able to operate a virtual reality wheelchair by moving their body as if it were controlling a standard joystick. The ability to operate a high-to-low dimensional map confirms the ability of the motor control system to exploit motor redundancy for reorganizing motor coordination [37,38,39].

A previous study on 15 subjects with thoracic SCI and 27 controls showed decreased FA and increased MD in SCI subjects bilaterally in the primary motor and sensory cortices supplying the lower limbs, the superior cerebellar cortex, medial prefrontal and anterior cingulate cortices, primary somatosensory and precuneus cortices and different regions in the corticospinal tracts, including the corona radiata, the posterior limb of the internal capsule, ventral pons and pyramids [9]. Although the number of subjects in our study was significantly smaller, a group comparison revealed similar white matter structures with significantly lower FA values in SCI participants. SCI participants had lower FA values than controls in the left hemisphere anterior corona radiata, cingulum and superior longitudinal fasciculus, together with the superior corona radiata in the right hemisphere. The corona radiata contains axons in the corticospinal tract leaving the cerebral cortex (originating in primary motor cortex M1, supplementary motor area SMA and primary somatosensory cortex S1) towards the posterior limb of the internal capsule, where they descend through the cerebellar peduncles to synapse on motor neurons located in the anterior horn of the spinal cord [40]. These same cortical structures have been shown to be involved in motor skill learning [41,42].

Our results show that 12 BMI training sessions over 4–6 weeks improved motor performance, upper-body ability and force production. These functional findings are consistent with earlier studies showing increased MMT and force output on cervical SCI participants practicing upper-body motions with a different BMI based on optical sensors and principal component analysis [43,44]. Here, we found that practice with the BMI also induced increases in FA values in one area that had shown lower FA in SCI subjects than controls, the left cingulum. The cingulum, part of the cingulate cortex, is a collection of white matter fibers wrapping around the frontal lobe to the temporal lobe directly superior to the corpus callosum and directly inferior the cingulate cortex. It carries connections to and from areas of the cingulate cortex, with fibers entering and exiting to various areas of association cortices [45] and projections from the supplementary motor area toward retrosplenial and parahippocampal regions [46]. This area was traditionally linked to emotion [47] and cognitive functions, such as attention, visual and spatial skills, working memory and general memory [48,49]. However, recent clinical and experimental findings suggest the cingulate cortex participates also in sensory and motor processes [50]. Electrical stimulation of the anterior cingulate cortex evokes skeletomotor responses [51] and complex coordinated movements involving large parts of the body, such as trunk leaning [52]. Neurons in the cingulum have been shown to exhibit task-related activity during both self-paced and sensory-triggered hand movements [53]. Positron emission tomography studies have demonstrated that the anterior cingulate cortex exhibits high metabolic activity in subjects performing cognitively-demanding stimulus-response tasks [54,55], and lesions of monkey’s posterior cingulate cortex have been shown to disrupt spatial learning [56].

These findings suggest the presence of changes in white matter localized to fibers connecting the motor and sensory cortices and both hemispheres, through circuits involved in motor skill learning, and these have not been reported earlier. Prior longitudinal studies of motor learning have reported changes in white matter in a primary motor cortex area corresponding to the hand representation after one week of motor adaptation [57], inferior to the intraparietal sulcus after six weeks of juggling [13], the frontal and parietal regions after six weeks of whole-body balancing training [14] and along the corticospinal tract and corpus callosum after nine9 sessions of BMI training over 3–4 weeks on unimpaired individuals [15]. Experience-dependent differences in FA have been reported previously, though the directions of FA differences are not consistent. Expert musicians have been shown to have higher FA values in the internal capsule compared to non-experts [58,59], whereas the corticospinal tract, internal and external capsule and inferior occipitofrontal fascicle of expert golfers have lower FA than non-experts [60]. Here, we show that the same protocol on SCI individuals resulted in increases in FA in the cingulum, even when the cingulum had shown decreased FA in SCI subjects when compared to controls. Changes in FA values have been shown to correlate with axon number and density and can be modulated by the addition and loss of myelin along the axonal sheath [61,62]. Moreover, a motor skill learning study on rats using both diffusion MRI and quantitative immunohistochemistry revealed increased FA and increased myelin staining in the motor cortex contralateral to the trained limb, and myelin staining density correlated significantly with learning rate [63]. Their results suggest that a novel motor skill induced structural changes in task-relevant white matter pathways, and those changes may reflect learning-related increases in myelination. Since the possibility of growth of new axons in 4–6 weeks is low, we suggest the structural changes observed in this study may also be due to changes in myelination patterns [15].

Our observations are different from previous results on control subjects, where BMI training-induced changes were localized to the right (non-dominant) hemisphere [15]. The authors suggest the non-dominant motor tracts in the right hemisphere were not as well tuned for the high level of control required to operate the BMI. Therefore, the largest improvements were expected from the left side of the body. Subjects in this study had their spinal cord injury 2–17 years ago. The BMI training promoted the use of motor functions that survived the injury, but that perhaps had not been exercised in a long time. This could explain the initial lower FA values on the left (dominant) hemisphere for the SCI population, where we observed training-induced changes in measures of white matter density.

### 4.1. Clinical Implications

We showed that five subjects with high-level spinal cord injury using a simple BMI for playing computer games for twelve 1.5-h sessions exhibited improvements in their upper-body ability and exhibited structural brain changes. Although brain structural changes were localized to small brain regions, these results are significant for the clinical impact of BMIs, since they suggest the possibility of long-term structural changes in brain connectivity related to the use of residual motor abilities after a few training sessions. Cortical atrophy following a spinal cord injury is a well-known complication [8,9,10], and several studies have proven that the sensory motor system undergoes functional reorganization after repeated practice of a motor task. Our results demonstrate that functional reorganization can still occur for people with SCI even in brain regions that show impairment and degradation. Learning-induced plasticity is relevant to the development of rehabilitative therapies and assistive devices that target the strengthening of neural and muscular resources that survived the injury [64,65]. In particular, plasticity is a key factor in guiding the coadaptation of closed-loop neural interfaces as the skill level of their users evolves with practice [66,67,68].

### 4.2. Limitations

Although subjects’ performance in their last session of center-out reaching and game-like activities was better than the first session, it seemed that the performance was still improving during the last sessions. This indicates that participants had not yet plateaued in their performance and could reach even higher levels of control with the BMI.

The design of this study lacked an additional clinical evaluation, and MRI was further removed (~6 weeks) from the end of training in order to evaluate the duration of the observed rehabilitative effects after BMI training. Motor impairment, weakness and muscular and cortical atrophy have been shown to progress further with lack of exercise after an SCI [5,6,7]. Therefore, we speculate that the reported increases in force and changes in the left cingulum would gradually decrease unless the participant maintains exercising his/her upper body. Having several additional clinical evaluations after the end of training could allow us to understand the rate of this potential degradation.

Unlike the maximum isometric force evaluation that showed significant improvements for various movements, we did not observe consistent improvements for any of the individual MMT movements. We believe this is due to each individual having a different initial level of impairment and, therefore, a unique potential for recovery.

In order to prevent spontaneous recovery as a confounding factor in observed improvements in motor function, only subjects that were in the chronic stage (>6 months post injury) were enrolled in this study. However, early SCI rehabilitation has been shown to dramatically enhance the recovery effects of therapy [69]. Future research will investigate the rehabilitative effects of BMI training in early stages after SCI. The rehabilitative potential of the BMI may also be beneficial for other types of neurological disabilities such as stroke, cerebral palsy or multiple sclerosis.

The design of this study lacked a control group with no training or one with a similar type of physical activity. However, several studies have already demonstrated the longitudinal reliability of DTI measures with control subjects that did not show FA changes [13,14,15]. Due to the nature of the training, we are unable to completely distinguish whether the observed changes in clinical measures of function and changes in FA originated from the remapping of body motion, or from the physical aspect of the training, or both. A previous study on unimpaired subjects found significant correlations between FA changes and performance changes, which suggest that changes in FA were related to learning [15]. Additionally, [70] showed that active motor learning affects both motor and sensory functions, while having the limbs displaced passively to simulate movements during training failed to induce sensory changes. This suggests that passive movement alone would not lead to the observed changes in the current study. However, it remains possible that an exercise program that targets the use of the remaining motor functions that survived the injury could result in similar changes. Moreover, a protocol that combines BMI use with traditional exercises for rehabilitation could potentially result in even stronger rehabilitative effects. The benefit of BMIs that use VR is that they can provide an extensive range of enjoyable environments where people can sustain the motivation to practice for extended periods of time [44,71]. Another benefit of this BMI is the ability to measure relatively small changes in movements that do not require a significant amount of effort by the subject. These small movements can be used for goal-directed activities, and the sensors can be easily repositioned as participants regain strength and mobility in one or more areas of the upper body. Moreover, this BMI can be used in combination with traditional exercise programs for rehabilitation of SCI.

Due to the small sample size (*n* = 5) of this study, the observed effects in the TBSS and ROI analysis would not survive corrections for multiple comparisons. A lack of a correction for multiple comparisons may increase the likelihood of finding false positives. However, three separate analyses (TBSS, ROI and tractography) showed increased FA in the left hemisphere cingulum.

The small sample size limited our ability to observe significant correlations between upper-body ability and BMI measures and changes in FA. There were only two MRI data points per participant (before and after). Therefore, the correlation analysis consisted of only 10 data points in total.

## 5. Conclusions

This BMI can provide severely impaired subjects with a powerful tool to enhance the proficient use of assistive devices, while promoting the reorganization process of both brain and body. A growing number of evidence indicates that movement reorganization while learning a new task occurs through plastic changes at different sites of the central nervous system [72,73], and this functional plasticity has been shown to be critical for motor rehabilitation [74,75]. This work demonstrates the strengthening of the neural and muscular resources that survived the spinal cord injury.

## Figures and Tables

**Table 1:** Participant characteristics.

Subject ID	Gender	Age (years)	Level of Injury	Time after Injury
S301	Male	35	C5–C6 Incomplete	15 years
S302	Male	43	C5 Complete	17 years
S304	Male	56	C6 Incomplete	16 years
S306	Male	31	C5 Incomplete	8 years
S307	Male	58	C5 Incomplete	2 years

**Table 2:** Spearman rank correlation coefficients (*r*) along with false discovery rate, FDR-corrected *p*-values between isometric force and BMI performance measures.

		Movement Time		Euclidean Error		Path Length		Dimensionless Jerk	
Right Shoulder	Flexion	0.311	(0.335)	0.400	(0.335)	0.332	(0.335)	0.267	(0.335)
	Protraction	0.583	(0.038) *	0.699	(0.015) *	0.565	(0.038) *	0.429	(0.110)
	Retraction	0.651	(0.017) *	0.454	(0.089)	0.671	(0.017) *	0.564	(0.038) *
	Horizontal abduction	0.463	(0.135)	0.440	(0.135)	0.470	(0.135)	0.362	(0.185)
	Extension	0.455	(0.118)	0.534	(0.118)	0.494	(0.118)	0.384	(0.158)
	Horizontal adduction	0.475	(0.134)	0.405	(0.134)	0.489	(0.134)	0.409	(0.134)
Left Shoulder	Abduction	0.543	(0.069)	0.449	(0.093)	0.583	(0.069)	0.511	(0.069)
	Protraction	0.428	(0.149)	0.555	(0.128)	0.456	(0.149)	0.351	(0.199)
	Retraction	0.779	(0.001)*	0.671	(0.006) *	0.786	(0.001) *	0.682	(0.006) *
	Extension	0.562	(0.058)	0.397	(0.143)	0.565	(0.058)	0.495	(0.081)

*r*-value (FDR-corrected *p*-value). * highlights values with *p* < 0.05.

**Table 3:** Spearman rank correlation coefficients (*r*) along with FDR-corrected *p*-values between isometric force and FA values in the left cingulum.

		Movement Time		Euclidean Error		Path Length		Dimensionless Jerk	
Right Shoulder	Left Cingulum FA	0.410	(0.240)	0.294	(0.410)	0.426	(0.220)	0.410	(0.239)

*r*-value (FDR-corrected *p*-value).

**Table 4:** Spearman rank correlation coefficients (r) along with FDR-corrected *p*-values between BMI performance measures and FA in the left cingulum.

		Left Cingulum FA	
Right Shoulder	Flexion	0.427	(0.220)
	Protraction	0.262	(0.464)
	Retraction	0.419	(0.228)
	Horizontal abduction	0.136	(0.707)
	Extension	0.164	(0.651)
	Horizontal adduction	0.171	(0.637)
Left Shoulder	Abduction	0.229	(0.525)
	Protraction	0.138	(0.703)
	Retraction	0.282	(0.429)
	Extension	0.195	(0.590)

*r*-value (FDR-corrected *p*-value).
